# Twenty-Five Years of Social Media: A Review of Social Media Applications and Definitions from 1994 to 2019

**DOI:** 10.1089/cyber.2020.0134

**Published:** 2021-04-09

**Authors:** Thomas Aichner, Matthias Grünfelder, Oswin Maurer, Deni Jegeni

**Affiliations:** ^1^ Department of Business Administration, John Cabot University, Rome, Italy.; ^2^ Johannes Kepler University Linz, Linz, Austria.; ^3^ Faculty of Economics and Management, Free University of Bozen-Bolzano, Bozen-Bolzano, Italy.

**Keywords:** social media, social networks, social media definition, social media applications, literature review

## Abstract

In this article, the authors present the results from a structured review of the literature, identifying and analyzing the most quoted and dominant definitions of social media (SM) and alternative terms that were used between 1994 and 2019 to identify their major applications. Similarities and differences in the definitions are highlighted to provide guidelines for researchers and managers who use results from previous research to further study SM or to find practical applications. In other words, when reading an article about SM, it is essential to understand how the researchers defined SM and how results from articles that use different definitions can be compared. This article is intended to act as a guideline for readers of those articles.

## Introduction

The term “social media” (SM) was first used in 1994 on a Tokyo online media environment, called Matisse.^1^ It was in these early days of the commercial Internet that the first SM platforms were developed and launched. Over time, both the number of SM platforms and the number of active SM users have increased significantly, making it one of the most important applications of the Internet.

With a similarly fast pace, businesses have moved their marketing interests toward SM platforms. The presence of both businesses and users on SM has further led to a shift in how companies interact with their customers, who are additionally no longer limited to a passive role in their relationship with a company.^2^ Customers give feedback, ask questions, and expect quick and customized answers to their specific problems. In addition, customers post text, pictures, and videos. Managers came to the understanding that the brand transition to SM ultimately involves a re-casting of the customer relationship, where the customer has become an ally or an enemy, not an audience.^3^

In research, SM is generally used as an umbrella term that describes a variety of online platforms, including blogs, business networks, collaborative projects, enterprise social networks (SN), forums, microblogs, photo sharing, products review, social bookmarking, social gaming, SN, video sharing, and virtual worlds.^4^ Given this broad spectrum of SM platforms, the applications of SM are quite diverse and not limited to sharing holiday snapshots or advertising and promotion.

As of January 2020, there are more than 110,000 publications that have the term “social media” in their title. Over the past 25 years in which these works were published, countless researchers have formulated quite varying definitions of SM—sometimes using alternative terms. In this period, the perceptions and understanding of what SM is, what it includes, and what it represents have also varied considerably. This can make it difficult for both researchers and companies to interpret and apply research findings; *for example*, when referring to SM in general, rather than referring to a specific type of SM, such as SN. It can be problematic to quote previous research that was carried out exclusively on one SM platform as being generalizable to SM, or to refer to results from research that defined SM as being more or less inclusive in terms of which platforms qualify as SM and which do not.

## Major Applications of SM

This section serves as the background of SM functions, rather than how the definition has changed. It provides a general, although not comprehensive, overview of some of the most important applications of SM over the past two and a half decades. This is important, as it highlights that SM cover a broad variety of scopes with specific functions and applications that can differ greatly between the different types of SM. Consequently, also the purpose and the users' perceived value of using SM varies. From a research perspective, this section serves as a foundation for classifying and discussing the SM definitions that are presented in the following chapters.

### Socializing with friends and family

Although not all SM platforms are specifically designed to facilitate socialization between its users, it may be considered one of the most apparent commonalities of all types of SM.^4^ Sometimes referred to as online communities, these SM platforms are valuable given that people often do not perceive a difference between virtual friends and real friends, as long as they feel supported and belong to a community of like-minded individuals.^5^ The SM helps to strengthen relationships through the sharing of important life events in the form of status updates, photos, etc., reinforcing at the same time their in-person encounters as well.^6^

The SM has also become a common tool for communication in families. A study conducted by Sponcil and Gitimu^7^ showed that for 91.7 percent of students the main reason for using SM is communicating with family and friends. In addition, 50 percent of the students communicated with their family and friends every day, and another 40 percent at least a few days a week. Williams and Merten^8^ suggest that by using SM in everyday life, people strengthen the relationships with family. Especially in relation to globalization and constant migration, it has become a vital tool for maintaining contact within migrant families. The need for transnational communication between family members and the people they left behind is of great importance.^9^

### Romance and flirting

Several studies suggest that SM significantly influences the romantic aspects of life. Aside from facilitating human interaction, communication technologies are also shaping and defining our relationships.^10^ It has been shown that SM is important in the starting phases of a relationship and has a significant influence on the relationship of many couples in the long run.^11^ The SM can help when starting a romantic relationship, *for example*, contacting a crush through SM can have special benefits for introverts, who otherwise would avoid face-to-face contact and would otherwise communicate less.^7^ Moreover, in some cases, online dating is preferable to live dating, as it gives the same feeling and allows users to avoid unnecessary discomforts.^11^ Finally, rejection on SM is less painful compared with face-to-face rejection.^10^ Further, users can contemplate their responses and do not have to worry about their physical appearance while conversing/chatting online, making it a less stressful environment to flirt with people on SM than face-to-face conversations.^12^

### Interacting with companies and brands

It is estimated that close to 100 percent of larger companies (both B2C and B2B) are using some sort of SM platform to inform their customers, gather information, receive feedback, provide after-sales service or consultancy, and promote their products or services. The key characteristic that makes SM so relevant for companies is the fact that SM allows for two-way communication between the brand and the customer.^13^ Sometimes referred to as “social customer relationship management,”^14^ SM can be viewed as an effective tool used to get closer to the customer. However, some studies suggest that what customers seek is somewhat different from what companies offer through SM.^14^ Customers are mainly interested in communicating easily and quickly with the company. From a business perspective, the company wants to make sure customers receive the right information in a timely manner, linking the customer closer to the brand and, simultaneously, controlling the flow of information. Successful SM managers understand how an SM platform works and is used by its customers, and they then develop corporate communication tools that fit the behavior of their users. Many researchers highlight the need for customer relationship management to adapt to the rise of SM^2^ to efficiently manage relationships with modern, connected, and empowered customers.

### Job seeking and professional networking

Another application of SM is to connect job seekers with employers. The vast majority of Fortune 500 companies use LinkedIn for talent acquisition.^15^ With more than 660 million users in 2020, it is an important tool for companies searching to expand their talent pool. This pool of individuals is extended, as the nature of SM also allows recruiters to identify and target, apart from active users, talented candidates who are passive or semi-passive and lure them to prospective job positions.^16^ In fact, through SM platforms such as LinkedIn, Facebook, and Twitter, recruiters can post job advertisements to lure potential applicants who are not actively looking for a job.^17^ Rather than the costly and time-consuming traditional ways of staffing with interviews and tests, hiring through SM offers recruiters the benefit of free access to prospects' profiles and an instant means of communication. For users, LinkedIn profiles allow them to create an idealized portrait by displaying their skills to recruiters and peers.^18^ Indeed, LinkedIn asks members to highlight their relevant skills, promoting their abilities and strengths, urging them to complete their profiles through getting recommendations and praise from peers/colleagues and clients for their performance or skills.^19^

### Doing business

The SM has a considerable impact on how companies approach clients and vice versa. In addition, SM utilizing SM as a means of understanding and informing customers has become imperative for businesses to remain competitive. The SM providers have created possibilities for companies to improve their internal operations and communicate in new ways with customers, other businesses, and suppliers.^20^ At the same time, companies can actively engage customers, encouraging them to become advocates of their brands.^2^ This is certainly important, as users can create online customer communities, which potentially add value to the brand beyond just increased sales.^20^ The engagement of customers can be beneficial, as they will frequently interact with the brand and share positive word-of-mouth since they have become more emotionally attached to the brand.^21^ This electronic word-of-mouth created in SM communities helps consumers in their purchasing decisions.^22^ This suggestion is important given that customers are actually more interested in other users' recommendations and word-of-mouth rather than the vendor-created product information.^23^

### Research questions

Reviewing the existent literature about SM applications inevitably leads to the question of whether the researchers had the same definition in mind when talking about SM, SN, online communities, and the like. It is also apparent that the focus of the researcher's interest has changed over time, and that the time when the research was conducted could have an impact on how the findings should be interpreted. Therefore, the remainder of this article aims at answering the following research questions (RQ):

***RQ1: How has the definition of social media changed from 1994 to 2019?***

***RQ2: What are the differences and commonalities in social media definitions from 1994 to 2019?***

## Method

**FIG. 1.**
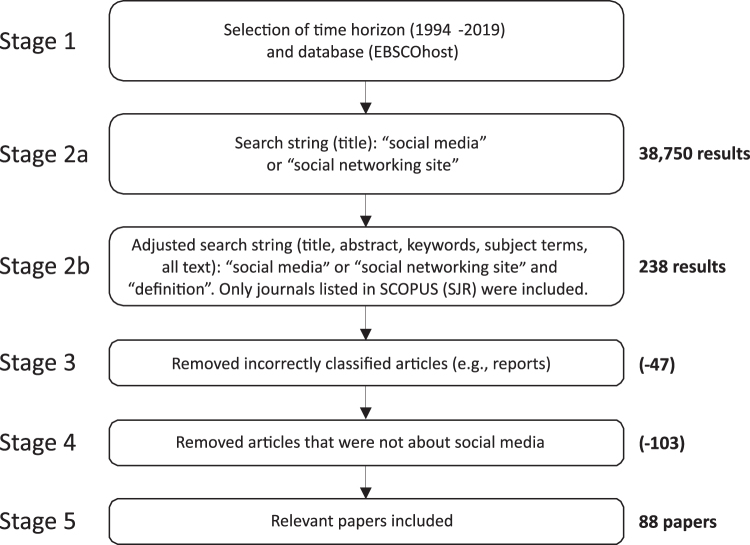
Structure and process of the systematic literature review.

To answer the two RQ, we decided to conduct a systematic literature review (SLR). Using a multi-step SLR approach as recommended by Tranfield et al.^24^ (Fig. 1), we structurally examined the literature between 1994 and 2019 to find all relevant SM definitions to identify the major differences and commonalities.

After identifying 88 potential papers, all the articles were read to find original definitions for SM or related terms. In addition, we used backward and forward snowballing, two methods frequently employed in academic research to find additional relevant sources based on the references used in the original publication (backward snowballing) and searched papers that cited the article (forward snowballing), respectively.^25^ In combination with the SLR, the backward snowballing led to the identification of a total number of 21 original definitions, including some definitions that were published in books and conference proceedings, which were not included in the SLR.

## Results

In this chapter, we present all major definitions of SM (and synonymous terms) that were formulated from 1994 to 2019 (Table 1). Table 1 further includes details about the source and the number of citations according to Google Scholar as of August 2020.

**Table 1.** Social Media Definitions with Author Names, Source, and the Number of Citations As of August 2020

Year	Definition	Authors	Source	Google scholar citations
1996	When computer networks link people as well as machines, they become social networks, which we call **computer-supported social networks** (CSSNs).	Wellman^26^	Annual Review of Sociology	1,886
1997	**Virtual communities** are groups of people who communicate with each other via electronic media and are a relatively new phenomenon.	Romm et al.^27^	International Journal of Information Management	384
1997	When a computer network connects people or organizations, it is a **social network**. Just as a computer network is a set of machines connected by a set of cables, a social network is a set of people (or organizations or other social entities) connected by a set of social relationships, such as friendship, co-working, or information exchange.	Garton et al.^28^	Journal of Computer-Mediated Communication	2,158
1999	**Virtual communities** are defined by bringing people together with a common set of needs or interests. Those needs or interests could span a variety of dimensions. Virtual communities could be organized around an area of interest (such as sports or stock investments), a demographic segment (certain age groups within the population), or a geographic region (metropolitan areas).	Hagel^29^	Journal of Interactive Marketing	3,325
2001	For the purposes of this article, we define a **virtual community** (in a relatively neutral way) as any entity that exhibits all of the following characteristics: (a) It is constituted by an aggregation of people. (b) Its constituents are rational utility-maximizers. (c) Its constituents interact with one other without physical collocation, but not every constituent necessarily interacts with every other constituent. (d) Its constituents are engaged in a (broadly defined) social-exchange process that includes mutual production and consumption (e.g., mutual dissemination and perusal of thoughts and opinions). Although each of its constituents is engaged in some level of consumption, not all of them are necessarily engaged in production. Such social exchange (as opposed to monetary or material exchange) is a necessary, but not always the only, component of interaction between the constituents of the entity. (e) The social interaction between constituents revolves around a well-understood focus that comprises a shared objective (e.g., environmental protection), a shared property/identity (e.g., a national culture or a lifestyle choice), or a shared interest (e.g., a hobby).	Balasubramanian and Mahajan^30^	International Journal of Electronic Commerce	699
2002	**Virtual communities** can be defined as groups of people with common interests and practices that communicate regularly and for some duration in an organized way over the Internet through a common location or mechanism. The location of the virtual community, although not physical, is important because it establishes the virtual “place” where the members meet. This location or mechanism may be a chatroom, bulletin board, or listserv e-mail program.	Ridings et al.^31^	The Journal of Strategic Information Systems	1,891
2005	SNSs [**social networking services**] are designed specifically to facilitate user interaction for a variety of goals, mainly dating, business networking, and promotion.	Marwick^32^	Conference: Association of Internet Res. 6.0	146
2006	At the most basic level, an **online social network** is an Internet community where individuals interact, often through profiles that (re)present their public persona (and their networks of connections) to others.	Acquisti and Gross^33^	Conference: Privacy Enhancing Technologies (PET)	2,680
2007	A **social networking site** (SNS) connects and presents people based on information gathered about them, as stored in their user profiles.	O'Murchu et al.^34^	Book: Viral Marketing: Concepts and Cases	263
2007	**Social network sites** are web-based services that allow individuals to (a) construct a public or semi-public profile within a bounded system, (b) articulate a list of other users with whom they share a connection, and (c) view and traverse their list of connections and those made by others within the system.	Boyd and Ellison^35^	Journal of Computer-Mediated Communication	19,908
2008	**Social networking sites** typically provide users with a profile space, facilities for uploading content (e.g., photos, music), messaging in various forms, and the ability to make connections to other people.	Joinson^36^	Conference: Proceedings of the SIGCHI Conference on Human Factors in Computing Systems	2,284
2009	**Social network sites** provide a public forum that enables the exchange of digital information, such as pictures, videos, text, blogs, and hyperlinks between users with common interests, such as hobbies, work, school, family, and friendship.	Sledgianowski and Kulviwat^37^	Journal of Computer Information Systems	668
2010	**Social media** is a group of Internet-based applications that builds on the ideological and technological foundations of Web 2.0, and that allows the creation and exchange of user-generated content.	Kaplan and Haenlein^38^	Business Horizons	19,656
2011	**Social media** is a honeycomb of seven functional building blocks: identity, conversations, sharing, presence, relationships, reputation, and groups.	Kietzmann et al.^39^	Business Horizons	5,174
2012	**Social networking sites** can be defined as virtual collections of user profiles that can be shared with others.	Hughes et al.^40^	Computers in Human Behavior	1,079
2013	A **social network site** is a networked communication platform in which participants (a) have uniquely identifiable profiles that consist of user-supplied content, content provided by other users, and/or system-level data; (b) can publicly articulate connections that can be viewed and traversed by others; and (c) can consume, produce, and/or interact with streams of user-generated content provided by their connections on the site.	Ellison and Boyd^41^	Book: The Oxford Handbook of Internet Studies	1,118
2015	**Social media** are Internet-based, disentrained, and persistent channels of masspersonal communication facilitating perceptions of interactions among users, deriving value primarily from user-generated content.	Carr and Hayes^42^	Atlantic Journal of Communication	386
2016	**Social media** is the colonization of the space between traditional broadcast and private dyadic communication, providing people with a scale of group size and degrees of privacy that we have termed “scalable sociality.”	Miller et al.^43^	Book: How the World Changed Social media	568
2018	For this study, we define “**social-media**” as Web sites and technological applications that allow its users to share content and/or to participate in social networking.	Leyrer-Jackson and Wilson^44^	Journal of Biological Education	17
2018	**Social media** is made up of various user-driven platforms that facilitate diffusion of compelling content, dialogue creation, and communication to a broader audience. It is essentially a digital space created by the people and for the people, and it provides an environment that is conducive for interactions and networking to occur at different levels (for instance, personal, professional, business, marketing, political, and societal).	Kapoor et al.^45^	Information Systems Frontiers	293
2019	For purposes of this chapter, we define **social media** as any online resource that is designed to facilitate engagement between individuals.	Bishop^46^	Book: Consumer Informatics and Digital Health	4

Before we assess the meaning and compare the definitions in terms of the two RQ, a few quantitative results are provided. Analyzing the 21 definitions, we found a lexical density (i.e., the percentage of nouns, adjectives, verbs, and adverbs) of 57.5 percent. The most frequently used word with 23 occurrences is “social,” followed by “people” with 12 occurrences, and “virtual,” “content,” “user,” and “network” with 8 occurrences each. In terms of two-word phrases, “social network[s]” (8 occurrences) is followed by “social media” and “social networking” (5 occurrences each), as well as “virtual communities” (VC) (4 occurrences).

Notably, the first formal definition is from 1996 and uses “computer-supported social networks” or “CSSNs,” although the term “SM” was coined about 2 years earlier. Later, researchers used different terms such as “virtual communities,” “social networks,” “social networking services,” “online social network,” “social networking sites,” “social network sites,” and “social media.” Although there are small variations in these terms, they can be classified into three categories: VC, SN, and SM. It is important to mention that all these definitions describe the same concept, but with different terms. Assessing the SM definitions that resulted from the SLR reveals that from 1997 to 2002, VC was the dominant term. In contrast, SN was used over a longer period, but it was dominant from 2005 to 2009. It was only in 2010 that researchers started using predominantly SM. But how did the definitions—independent from their terminology—change?

Throughout the observed period, the role of SM, as an enabler for human interaction as well as an avenue to connect with other users, has been a constant in defining SM. In early definitions, the focus was mainly on people and how people interact, whereas later definitions (after 2010) have largely substituted the term “people” with “user” and placed more focus on generating and sharing content. This changed focus, with regard to both the application of SM and the terminology of people versus user, may also reflect the increasingly important role of anonymity in SM.^47^

The role of user-generated content is not reflected in early definitions, whereas it has become a central part of recent definitions. It was Kaplan and Haenlein^38^ who first mentioned “creation,” whereas later definitions use terms such as “user-supplied content” and “user-driven platforms” in addition to “user-generated content,” which is the common term used in research and practice today.

Another notable change is that until 2009, several researchers included the common interests that linked people with each other, whereas this link is completely missing in post-2010 definitions. Again, this may be reflected by the fact that in the early days, SM users were mostly close or loosely related friends communicating with each other, whereas in recent years, SM has evolved to a set of media that are also used as a powerful tool by companies, celebrities, and influencers to reach the masses.^48^

Finally, although sharing information and content is generally not the central aspect in defining SM, the terminology has changed over time. Until 2010, researchers used “exchange” or “upload,” which were substituted with the term “share” in subsequent years. The underlying meaning, however, remained the same.

## Conclusions

About 60,000 articles have cited the SM definitions summarized in this article. Therefore, the value this research provides goes beyond a simple overview of the definitions and major applications of SM in the 25 years, since the term was originally coined. The result is a timeline of SM definitions that helps researchers and practitioners to quickly put the results of previous research in perspective and to avoid time-consuming research of the single definitions in different papers. Why is this necessary? This is because, based on the definition, the results may need to be interpreted in a more or less different way.

One notable result is that, although SM is one of the main research areas in social sciences (and beyond) and its landscape has been changing quickly, only a handful of scholars have made an effort to develop a definition of SM. Although some elements, *for example*, the fact that SM connects people, are common, the definitions are rather different from each other. The commonalities and differences highlighted in the previous section allow for the division of the definitions into two main streams: those published before 2010 and after 2010. Before 2010, SM was commonly approached as a tool of connectivity for people with common interests. After 2010, the focus changed to creating and sharing user-generated content.

These results are in line with previous research about the evolution of SM literature, which concluded that SM definitions changed over time, namely from platforms for socializing in the past to tools for information aggregation.^45^ Similarly, Kapoor et al.^45^ found that there was an evolution in SM definitions and a cut in the early 2010. Our research shows that there is no single or commonly accepted definition, but that several definitions have been co-existing and found broad acceptance in literature.

Future SM researchers can use these findings to better compare SM articles and avoid flaws in their theory or methodological design. Especially when comparing the results of empirical studies, it may be critical to consider both when the study was conducted and which SM definition was used as a basis for hypothesis development and data analysis. In addition, this article gives SM researchers the possibility to make an informed choice of which SM definition to use for their studies.

Given the method employed to identify the SM definitions, we are confident that this is the most comprehensive overview that includes all major publications. However, the results may be limited by the original search terms used to identify the papers to be included in the SLR. Although the use of backward snowballing should have helped in minimizing this risk, there may still be some less explicit definitions of SM that were not included in this article. In addition, non-English articles and other gray literature were not considered, which is common criticism in academic research. Future research could also try to identify the differences in how SM is defined by researchers from different scientific backgrounds, *for example*, marketing versus medicine versus psychology versus anthropology versus engineering versus information technology. It would also be insightful to see whether there are tendencies of certain researchers, *for example*, from engineering, to base their research on specific definitions rather than on others. For example, if one definition is dominant in engineering but not in medical research, this would imply that interdisciplinary research about SM applications needs to be compared more carefully, as the basic definition differs. Similarly, it would be interesting to link the use of SM definitions to the cultural or national context of where the research was carried out, *for example*, to identify whether European versus American versus Asian researchers have a generally different understanding of SM and its applications. These possible cultural differences in the definition or selection of an SM definition as a basis for research could be linked to the fact that in different countries and cultural clusters different SM platforms are more or less popular.^49^ Overall, our research will help compare findings from SM literature more easily and avoid misinterpretations of past and future research.
